# Rationale and methods of a randomized clinical trial to compare specific exercise programs versus home exercises in patients with subacromial impingement syndrome

**DOI:** 10.1097/MD.0000000000016139

**Published:** 2019-07-26

**Authors:** Héctor Gutiérrez-Espinoza, Felipe Araya-Quintanilla, Jonathan Zavala-González, Gonzalo Gana-Hervias, Vicente Martínez-Vizcaino, Celia Álvarez-Bueno, Iván Cavero-Redondo

**Affiliations:** a Faculty of Health, University of the Americas; b Physical Therapy Department, Clinical Hospital San Borja Arriaran; c Physical Therapy School, University Gabriela Mistral; d Adult Orthopedic Department, Clinical Hospital San Borja Arriaran, Santiago, Chile; e Universidad de Castilla-La Mancha, Health and Social Research Center, Cuenca, Spain; f Universidad Autónoma de Chile, Faculty oh Health Sciences, Talca, Chile.

**Keywords:** exercise therapy, home exercises, kinematics alterations, randomized clinical trial, subacromial impingement syndrome

## Abstract

**Background:**

Subacromial impingement syndrome (SIS) is a common clinical condition with a multifactorial etiology. Currently, there is a great variety of therapeutic exercise modalities aimed at treating SIS. Based on alterations of the glenohumeral and/or dysfunctional scapular kinematics associated with SIS, we hypothesize that the implementation of a specific exercise program with special focus on the correction of muscle deficits should be more effective than nonspecific exercises performed at home. This article describes the rationale and methods of study aimed at testing the effectiveness of specific exercise programs versus home exercises in patients with SIS.

**Method/Design:**

Ninety-four patients between the ages of 18 and 50 years referred to the Physical Therapy department of the Clinical Hospital San Borja Arriaran in Chile will be randomized to 2 treatment arms. The intervention group will receive a program of specific exercises with a duration of 12 weeks, taking as a reference the algorithm of clinical decision proposed by a panel of experts. The control group will receive a program of nonspecific exercises to perform at home. Three evaluations will be performed: before the initiation of treatment, and at the 12-week and 1-year follow-up. The primary outcome measure will be the shoulder function by the Constant-Murley questionnaire, and the secondary outcome measures will be the upper extremity function by the Disabilities of the Arm, Shoulder and Hand questionnaire, and pain by the visual analog scale.

**Discussion:**

This article reports the design of a randomized clinical trial aimed at assessing the effectiveness of a specific exercise program versus home exercises in patients with SIS.

**Trial registration:**

Brazilian registry of clinical trials UTN number U1111-1204-0268. Registered 27 September 2017.

## 1 Introduction

Subacromial impingement syndrome (SIS), also called external impingement,^[1]^ is defined as a mechanical compression or abrasion of the supraspinatus tendon, subacromial bursa, or long head of the biceps tendon beneath the anterior undersurface of the acromion, coracoacromial ligament, or the acromioclavicular joint during elevation of the arm.^[2]^ It is a very common clinical condition, and represents between 50% and 86% of all consultations for shoulder pain in primary care,^[3–5]^ and 36% in secondary care.^[6]^ Three progressive stages have been defined for this syndrome. In stage 1, a reversible edema and hemorrhage are presented, in patients typically younger than 25 years. In stage 2, chronic inflammation, defined by histological changes such as fibrosis and tendinitis due to repeated mechanical irritation, is usually observed in patients aged 25 to 40 years. Stage 3 typically occurs in individuals older than 40 years with an extended history of shoulder pain with osteophytes or glenohumeral osteoarthrosis, and a partial or full-thickness rotator cuff tear.^[2]^

Currently, SIS is considered a multifactorial condition for which that etiology has been explained by intrinsic and extrinsic mechanisms of rotator cuff pathology.^[7,8]^ The extrinsic factors explain the external mechanical compression of the anatomical structures that are within the subacromial space^[8]^: anatomical factors such as variation in the type and shape of the acromion, the acromioclavicular joint and the thickening of the coracoacromial ligament and biomechanical factors such as alterations in posture, loss of extensibility of the posterior capsule and the pectoralis minor muscle, and alterations in the glenohumeral and scapulohumeral kinematics associated with deficits in the muscular performance of the rotator cuff and the scapular muscles.^[7,8]^

The association between alterations of the glenohumeral and scapular kinematics and SIS has been previously established.^[9–15]^ A meta-analysis found that the pattern of scapular kinematics alterations is characterized by a reduction of the upward and external scapular rotation, and an increase in clavicular elevation and retraction, when compared with healthy asymptomatic subjects.^[11]^ Regarding glenohumeral kinematic changes, a published study showed that patients with SIS only have higher elevation of the humerus in the scapular plane at 30° and 60°, and an increase of the anterior translation of the humeral head between 90° and 120° of anterior flexion, when compared with healthy asymptomatic subjects.^[15]^ These kinematic alterations have been associated with a deficit in the function of some muscles, although there is no consensus on the results of the studies; at the scapulohumeral level, the most significant changes are an increase in the activity of the upper trapezius, a reduction in the activity of the serratus anterior and the inferior trapezius; and a delay in the activation time of the serratus anterior and inferior trapezius.^[9,10,16–19]^ At the glenohumeral level, there is a reduction in the activity and coactivation of the rotator cuff, especially the infraspinatus and subscapular in the first phase of the arm elevation movement.^[7,8,10,16,19]^

Several modalities of exercise have been used for the management of the SIS.^[20–22]^ Previous reviews have shown that therapeutic exercise decrease the pain and improve the function,^[20–26]^ but there is no exercise protocol considered as *“reference standard”* for the conservative treatment of patients with SIS.^[20,21,23,24]^ A consensus decision algorithm for physiotherapists based on clinical reasoning to evaluate and treat patients with shoulder pain have been proposed, in which the selection and prescription of exercises is made based on clinical findings rather than the structural pathology.^[27]^ The specific exercises are the fundamental basis of the treatment, because it seeks to correct muscle deficits and thus restore alterations of the glenohumeral or scapular dysfunctional kinematics associated with SIS.^[22,27]^

This article reports the rationale and methods of a trial aimed to assess the effectiveness in the short- and long term of a program of specific exercises based on the algorithm of clinical decision proposed by a panel of experts, on functional improvement and pain relief compared with exercises at home in patients aged between 18 and 50 years with stage 2 SIS.

## 2 Method

### 2.1 Study design/setting

**Figure 1:**
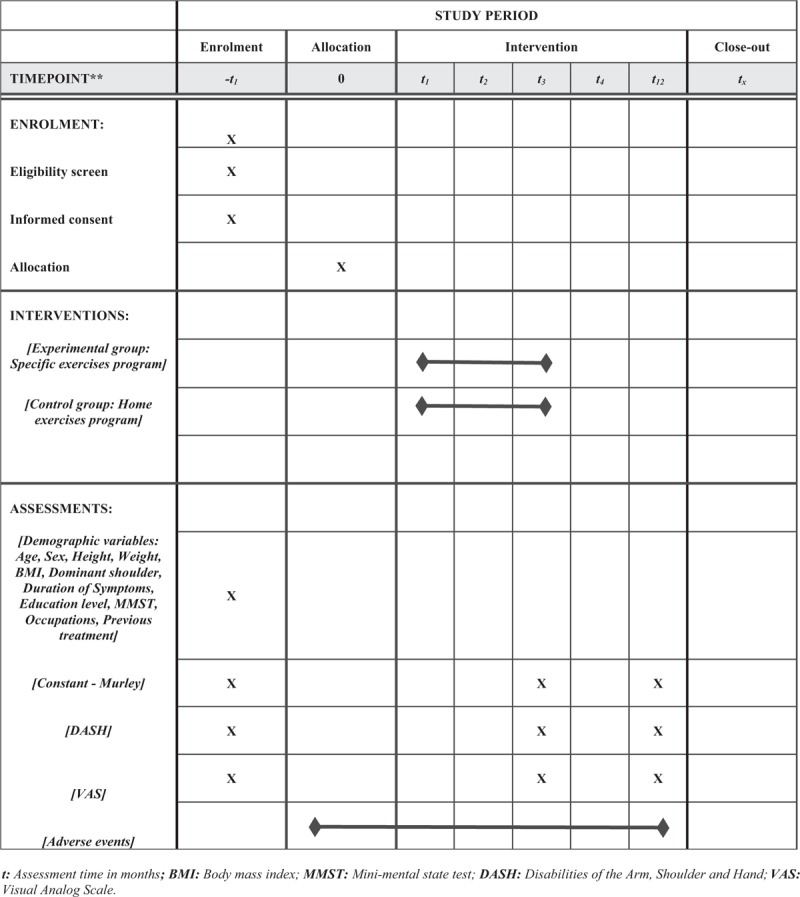
Standard protocol items: Recommendations for Interventional Trials (SPIRIT) figure. BMI = Body mass index, DASH = Disabilities of the Arm, Shoulder and Hand, MMST = Mini-Mental State Test, VAS = visual analog scale.

This will be a study single-blinded, randomized controlled trial with 2 parallel groups. It will be conducted at the Physical Therapy Department of the Clinical Hospital San Borja Arriaran in Chile. This protocol was written and based on Standard Protocol Items: Recommendations for Interventional Trials guidelines.^[28]^ The participants are residents who will mainly be recruited in the city of Santiago. The participants will be informed about the research, procedures, risks, and benefits by HG-E (author of this protocol). If they agree, they will sign an informed consent form. Only those participants who read and agree to the protocol and who sign the informed consent form will be part of the study, following the schedule described in Figure 1.

### 2.2 Participants

Ninety-four adult patients with stage 2 SIS based on Neer classification,^[2]^ with a poor response to the initial conservative treatment, and who were under evaluation for surgery, will be included. The diagnosis will be performed by an orthopedic surgeon based on the clinical history according to the following diagnosis criteria: pain located on the anterolateral side of the shoulder for at least 6 months and poor response to initial conservative treatment (at third month). Clinical signs of SIS, such as painful arc during active elevation of the arm, positive Neer or Hawkins-Kennedy test, and pain to the resisted external rotation, to the abduction or positive Empty Can test. These clinical signs have shown sensitivity and specificity values >74% for the diagnosis of SIS.^[29]^ Furthermore, to confirm the exclusion of the other shoulder pathologies, the clinical diagnosis will be complemented with imaging studies,^[30]^ which will include anteroposterior, axial, and outlet radiography, soft tissue echotomography and nuclear magnetic resonance. All patients will be treated with subacromial steroid injection of 40 mg methylprednisolone and 500 mg of oral naproxen twice daily for 14 days; after 2 weeks, they will be referred to the Physical Therapy Department.

#### 2.2.1 Inclusion criteria

To participate in the study, the patients will have to fulfill the following inclusion criteria: older than 18 years old, and referred by the Adult Orthopedic Department with a clinical and imaging diagnosis of stage 2 SIS, and with poor response to initial conservative treatment and accept and sign the informed consent.

#### 2.2.2 Exclusion criteria

Patients will be excluded if they meet the following criteria: presenting pathologies of cervical origin (cervical radiculopathy, etc), other pathologies of the shoulder joint complex, such as osteoarthritis in the acromioclavicular or glenohumeral joints, calcific tendinitis, adhesive capsulitis, and glenohumeral instability; stage 3 SIS (partial or full-thickness tear rotator cuff); a history of acute trauma, previous surgery, or a previous fracture in the affected shoulder; have previously been infiltrated with corticosteroids in the affected shoulder (6 months); and some degree of cognitive impairment scoring <26 points on the Mini-Mental State Test (MMST).

### 2.3 Interventions

The intervention group will receive a specific exercise program based on motor training, taking as a reference the clinical decision algorithm proposed by a panel of experts.^[27]^ In the initial stage, conscious muscular control is necessary to improve proprioception and normalize the rest position scapular,^[22,31]^ for which Mottram et al^[32,33]^ proposed the *“Scapular orientation exercise*.*”* Therefore, 3 scapular control exercises are continued: movement of bilateral anterior flexion of the shoulders up to 60°,^[34,35]^ closed kinetic chain exercise called *“Unilateral bench press*,*”*^[36]^ and scapular control exercise with bilateral shoulder retraction and extension in prone position.^[37]^ Finally, 2 glenohumeral control exercises are prescribed to restore centralization and prevent superior translation of the humeral head: isometric external rotation performed with shoulder adduction^[38]^ and isometric adduction exercise of the shoulder on the scapular plane at 30° and 60°.^[39]^ The exercises should not produce pain; only mild to moderate pain levels (<4/10 on the visual analog scale) are accepted after the session, and a maximum of 4 exercises per session. Dose will be related to the goal of each exercise and will be adjusted in relation to the individual patient, 8 to 10 repetitions of each exercise will be performed, with 5 to 10 seconds of task maintenance and 30 seconds to 1 minute of rest between each repetition. The criteria for exercise progression will be good-quality shoulder movement, and minimal pain increase during exercises. There will be 3 weekly sessions for 12 weeks.

In the control group, all patients will attend an appointment with a physiotherapist, where they will be aware of their clinical condition and will be provided with a printed document with a program of nonspecific exercises to perform at home. This program consists of 6 exercises without external load for the shoulder and cervical spine; movements of shoulder abduction in the frontal plane, shoulder retraction, elevation of the shoulder, retraction of the cervical spine, passive stretching of the upper trapezius, pectoralis major, and minor muscles.^[40,41]^ This will be performed twice a day for 12 weeks. To monitor adherence to treatment, patients will be contacted by phone in the fourth and eighth weeks of treatment.

### 2.4 Outcome measures

Baseline and postintervention outcome variables and potential confounders will be measured in both the intervention and control groups. Measurements will be performed before starting the treatment, at the end of twelfth week, and at the 1-year follow-up.

#### 2.4.1 Primary outcome measure

Function of the shoulder, as measured by the Constant-Murley questionnaire, will be the primary outcome measure.^[42,43]^ This consists of 4 subscales: 2 based on an interview with the patient regarding pain (a maximum of 15 points will be assigned) and activities of daily living (20 points) and the other 2 based on physical examination about active range of movement (40 points) and muscle strength (25 points), to a maximum score of 100 points.^[42]^ This questionnaire presents a high correlation with other scales and shoulder-specific questionnaires, also showing a high reliability and sensitivity to detect postintervention changes in a wide variety of shoulder pathologies.^[44]^

#### 2.4.2 Secondary outcome measures

Upper extremity function, as measured by the Disabilities of the Arm, Shoulder, and Hand questionnaire, is the secondary outcome measure.^[45]^ This is a self-administered questionnaire with 30 items related to the degree of difficulty during the previous week when performing various physical activities due to problems in the shoulder, arm, or hand (21 items); the severity of each of the symptoms, pain related to activity, tingling, weakness, and rigidity (5 items); and the effect of the problem on social activities, work, sleep, and psychological impact (4 items). A rating scale is used that ranges from 1 (without difficulty to perform, without symptoms, or without impact) to 5 (unable to do, very severe, or high impact symptom); the final score ranges from 0 to 100 (the higher the score greater disability). A transcultural adaptation has been made to the Spanish language, whose version showed excellent results with respect to validity, reliability, and sensitivity to change.^[46]^

Pain intensity at rest and movement will be assessed using the visual analog scale, which consists of a horizontal line 10 cm in length, where the left end represents the 0 or *“painless”* and the right end the 10 or *“worst pain imaginable.”* The patient is asked to mark with a vertical line the magnitude of the pain he feels at the time of the evaluation. It is a 1-dimensional, simple, and reproducible assessment method.^[47]^

#### 2.4.3 Potential confounders

Clinical variables: dominant shoulder affected, duration of symptoms (months), and previous treatments received in the last 3 months (supervised physical therapy, only exercises, use of paracetamol/nonsteroidal anti-inflammatory drugs, and use of opioids) will be evaluated.

Anthropometry and body composition: weight will be measured with the patient barefoot and in light clothing. Height will be measured using a wall stadiometer, with the patient barefoot and upright and with the sagittal midline touching the back board. Body mass index will be calculated as weight in kilogram divided by the square of the height in meters.

##### 2.4.3.1 Socioeconomic status

Educational level will be classified as primary education (functionally illiterate, without any studies, or those not completed primary education), middle education (primary education, high school/secondary education or baccalaureate) and university education (college or PhD degree). Occupation will be categorized as heavy load, light load, and sick leave in the last month.

The cognitive status of all patients will be evaluated using the MMST.^[48]^ This is the most commonly used test for standardized cognitive assessment in the clinical setting.^[49]^ The scores that patients with mild-to-moderate cognitive impairment have for this questionnaire are influenced by a number of sociodemographic variables, such as age and educational level.^[50,51]^ The cut-off scores are, however, variable,^[52]^ we set them at 26/27 in our study to make sure that patients could follow simple commands and could also follow therapeutic indications for their treatments.

### 2.5 Sample size calculation

Sample size for this trial is based on an expected mean difference between groups of 17 points of the Constant-Murley questionnaire, which is the minimum clinically important difference.^[53]^ The mean assumed for the calculation was 52.5 with a standard deviation of 23 points based on results of other randomized clinical trials.^[40]^ To detect this difference between both treatments, with a value of α = 0.05 (probability of committing a type I error) and a statistical power of 90%, a minimum of 39 patients per group is needed. This minimal sample size estimate has been increased by 20% after considering the potential dropouts, totaling 47 patients for each group. According to this, the experimental hypothesis proposed is that there will be a difference of at least 17 points in the Constant-Murley questionnaire in the intervention versus control group that will follow a program of exercises at home. Sample size was performed using the Stata SE software, version 15 (StataCorp, College Station, TX).

### 2.6 Recruitment

Recruitment of the participants began in September 2017 and is expected to finish in January 2020. Before inviting participants to sign the informed consent, information on the study goals and procedures will be provided verbally. The participants will be also invited to raise questions or doubts on any aspect of the study. Data confidentiality guarantees will be provided to participants by the principal investigator. Written consent will be obtained from all participants before registration, and participants may withdraw from the trial at any point in time without penalties. The written consent form includes information regarding the background and purpose of the study, therapeutic interventions, outcomes, and the expected benefits and drawbacks.

### 2.7 Randomization and blinding

**Figure 2:**
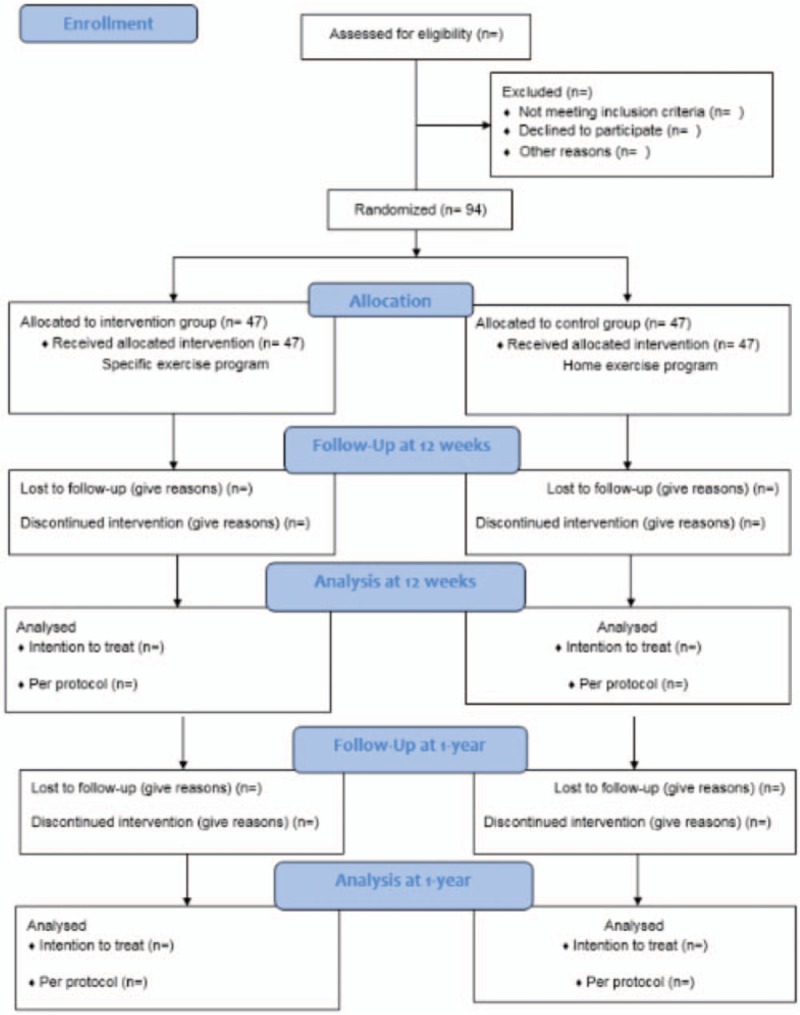
Flow diagram of patients through phases of clinical study.

Participants will be allocated to the 2 groups in a random manner through a sequence of numbers that will be generated by a computer program before beginning the selection process. The group assigned to each patient will be kept in a sealed envelope with the objective of concealing the assignment to the researcher, who will decide on the entry of subjects to the study (Fig. 2). Given the nature of the therapeutic interventions studied, physiotherapists and patients, blinding will be not possible. The evaluator and statistician, however, will not know to which group the subjects evaluated will belong.

### 2.8 Data management

Information obtained from the evaluation of each participant will be recorded on a paper print-out. The information will then be hand written on a paper document case report form and entered into an Excel file for future statistical analyses. In accordance with the Personal Information Protection Act, the names of all participants will not be disclosed and a unique identifier number given during the trial will be used to identify participants. All of the participants will be informed that the clinical data obtained in the trial will be stored in a computer and will be handled with confidentiality. The participants’ written consent will be stored by the principal investigator.

### 2.9 Statistical analysis

The continuous variables will be presented as means and standard deviations, and the categorical ones as number and percentage; to determine whether parametric statistical tests are appropriate for use in the analysis of the data, the fitting to normal distribution will be evaluated using both statistical (Shapiro-Wilk test) and graphical (normal probability plot) methods. To examine baseline differences of 2 groups, a 1-way analysis of variance (for continuous variables) and chi-square tests (for categorical variables) will be conducted. Repeated measures analysis of covariance will be performed on outcome variables with time (3 levels: preintervention, postintervention, and 1-year postintervention) as the within-subject factor and with type of intervention (2 levels: specific exercise program vs home exercises). The covariates were baseline values of each outcome variable.

Data will be processed independently by 2 researchers, and inconsistencies will be detected using the VALIDATE command of Epi Info (CDC) software. After checking the truthfulness of outliers and extreme values, these will be winsorized using below the 1st percentile and above the 99th percentile of the distribution of variables. Before conducting the study, after considering the nature of the missing data in those patients with incomplete entries, these will be imputed using chained equations.

### 2.10 Harms

To collect, assess, report, and manage the potential adverse effects of the interventions that will be performed in the study, at the beginning and end of each treatment session, patients from both groups will have a logbook available. According to the informed consent that patients will sign when they are recruited for the study, patients who show an increase in symptoms after 48 hours of the session will require an immediate evaluation by an orthopedic surgeon.

### 2.11 Ethics

The study will be conducted under the Declaration of Helsinki principles,^[54]^ and the norms of good clinical practice. The study protocol has been approved by the Ethical Committee of the Central Metropolitan Health Service of Chile with the reference number 048975. This research was registered in the Brazilian Registry of Clinical Trials with the number U1111-1210-3555.

## 3 Discussion

The aim of this article was to describe the rationale and methods of a randomized controlled trial aimed to test the effectiveness of 2 exercise programs in patients with stage 2 SIS, with poor response to initial conservative treatment, who were under evaluation for surgery. The intervention group will receive a program of specific exercises, taking as reference the clinical decision algorithm proposed by a panel of experts.^[27]^ For its part, the control group will receive a program of nonspecific exercises to perform at home.

There are several modalities of exercise used in the management of SIS.^[20–22,26]^ Therapeutic exercise has been shown to reduce pain and improve functional loss associated with SIS;^[20–26]^ however, specific components of the exercise protocol are unknown.^[20–22,24,26]^ A systematic review showed that programs including a combination of scapular stabilization exercises, rotator cuff resistance exercises, active exercises to improve range of motion, and muscle stretching exercises were effective in the management of SIS.^[24]^ Nevertheless, the evidence is limited, making it difficult to establish which type of exercise is more clinically effective.^[24–26]^

Based on the alterations of glenohumeral and scapular kinematics associated with the SIS, the expert consensus has recommended including specific exercises to treat muscle function deficits, which can be subdivided in neuromuscular control deficits (muscle cocontraction and force couples) and muscle strength deficit, which also includes an imbalance in muscle activity and an alteration of muscle activation times.^[22,31,55]^ Another recently published systematic review, however, concluded that there is insufficient evidence to support or refute the effectiveness of specific exercises used in the conservative treatment of patients with SIS.^[56]^

The general principles of our exercise protocol are exercises should be performed with optimal scapular positioning and control without abnormal compensatory trunk movement; start with low load/low activation exercises, without pain, with the arms below the shoulders level, emphasizing the quality of the performance of the motor task, performed slowly, consciously, and progressively; progress to prone and lateral decubitus exercises that increase the load, selectively activating weak muscles such as serratus anterior and inferior trapezius, with minimal activation of overactive muscles such as upper trapezius and deltoids, avoiding muscle fatigue; and decrease in feedback gradually during the course of the exercise, until the realization of exercises subconsciously and automatically.^[27,31,55]^

To strengthen the reliability of the results, important methodological factors have been considered planning this study. To avoid selection bias, a clinical and imaging diagnosis of the SIS was considered, including echotomography and magnetic resonance imaging; participants will be randomly assigned to the groups through a hidden allocation sequence. Furthermore, adjusting the sample size for possible losses or dropouts and increasing the number of patients recruited by 20% was considered; in the event of loss or withdrawals, statistical analysis will be carried out by protocol and intention to treat. To minimize measurement bias, all evaluations will be performed by 2 physiotherapists external to the research team, who will also remain blinded in relation to the treatment groups; the statistician will remain blinded to the group assignment of the participants; The outcome measures used are suitable and frequently used in clinical practice, as well as having good levels of validity and reliability. The 1-year follow-up will allow the effectiveness of the therapeutic interventions to be established in the long term.

Our study has some limitations. One important limitation is that the clinical decision algorithm proposed by a panel of experts has not yet been validated. Finally, the blinding of physiotherapists and patients was not achievable given the nature of the interventions studied.

To the best of our knowledge, this is the first clinical trial that compares a specific exercise program based on a clinical decision algorithm proposed by a panel of experts, with a program of nonspecific exercises performed at home. The results of this study will add evidence to the limited and controversial body of knowledge related to the effectiveness of the different modalities of therapeutic exercises that are prescribed for patients with SIS.

## Acknowledgments

The investigators would like to thank Mrs Hernan Cañon Jones for her administrative support at our investigation.

## Author contributions

HGE is responsible for study design, clinical protocols, ethical approval, clinical administration of the study, data collection, manuscript drafting, and editing. FAQ and JZG are responsible for study design, clinical protocols, clinical administration of the study, data collection, manuscript editing, and review. GGH is responsible for study design, clinical protocols, clinical and scientific supervision, manuscript editing, and review. VMV, CAB, and ICR are responsible for study design, clinical protocols, clinical and scientific supervision of manuscript drafting, editing, and review. All authors read and approved the final manuscript.

**Conceptualization:** Héctor Gutiérrez-Espinoza, Felipe Araya-Quintanilla, Gonzalo Gana-Hervias, Vicente Martínez-Vizcaíno, Celia Álvarez-Bueno, Iván Cavero-Redondo.

**Data curation:** Vicente Martínez-Vizcaíno.

**Formal analysis:** Gonzalo Gana-Hervias, Vicente Martínez-Vizcaíno, Iván Cavero-Redondo.

**Investigation:** Héctor Gutiérrez-Espinoza, Jonathan Zavala-Gonzalez, Gonzalo Gana-Hervias.

**Methodology:** Héctor Gutiérrez-Espinoza, Felipe Araya-Quintanilla, Jonathan Zavala-Gonzalez.

**Project administration:** Héctor Gutiérrez-Espinoza, Gonzalo Gana-Hervias, Celia Álvarez-Bueno.

**Supervision:** Felipe Araya-Quintanilla, Jonathan Zavala-Gonzalez, Gonzalo Gana-Hervias, Iván Cavero-Redondo.

**Validation:** Iván Cavero-Redondo.

**Visualization:** Jonathan Zavala-Gonzalez.

**Writing – original draft:** Héctor Gutiérrez-Espinoza, Felipe Araya-Quintanilla, Vicente Martínez-Vizcaíno, Celia Álvarez-Bueno, Iván Cavero-Redondo.

**Writing – review and editing:** Héctor Gutiérrez-Espinoza, Vicente Martínez-Vizcaíno, Celia Álvarez-Bueno, Iván Cavero-Redondo.
